# An interpretable CT-based machine learning model for predicting recurrence risk in stage II colorectal cancer

**DOI:** 10.1186/s13244-025-02009-2

**Published:** 2025-07-31

**Authors:** Ziqi Wu, Liya Gong, Jingwen Luo, Xiaobo Chen, Fan Yang, Junyan Wen, Yanyu Hao, Zhishan Wang, Ruozhen Gu, Yuqin Zhang, Hai Liao, Ge Wen

**Affiliations:** 1 Department of Medical Imaging, Nanfang Hospital, Southern Medical University, Guangzhou, China; 2 Department of Radiology, Guangdong Provincial People’s Hospital (Guangdong Academy of Medical Sciences), Southern Medical University, Guangzhou, China; 3 Guangdong Provincial Key Laboratory of Artificial Intelligence in Medical Image Analysis and Application, Guangzhou, China; 4 The First School of Clinical Medicine, Zhejiang Chinese Medical University, Zhejiang, China; 5 Department of Radiology, Guangxi Medical University Cancer Hospital, Nanning, China; 6 KnowX Tech Inc., Toronto, ON Canada; 7 Department of Radiology, The Affiliated Lihuili Hospital of Ningbo University, Ningbo, China

**Keywords:** Colorectal cancer, Computed tomography, Machine learning, Radiomics, Prognosis

## Abstract

**Objectives:**

This study aimed to develop an interpretable 3-year disease-free survival risk prediction tool to stratify patients with stage II colorectal cancer (CRC) by integrating CT images and clinicopathological factors.

**Methods:**

A total of 769 patients with pathologically confirmed stage II CRC and disease-free survival (DFS) follow-up information were recruited from three medical centers and divided into training (*n* = 442), test (*n* = 190), and validation cohorts (*n* = 137). CT-based tumor radiomics features were extracted, selected, and used to calculate a Radscore. A combined model was developed using artificial neural network (ANN) algorithm, by integrating the Radscore with significant clinicoradiological factors to classify patients into high- and low-risk groups. Model performance was assessed using the area under the curve (AUC), and feature contributions were qualified using the Shapley additive explanation (SHAP) algorithm. Kaplan–Meier survival analysis revealed the prognostic stratification value of the risk groups.

**Results:**

Fourteen radiomics features and five clinicoradiological factors were selected to construct the radiomics and clinicoradiological models, respectively. The combined model demonstrated optimal performance, with AUCs of 0.811 and 0.846 in the test and validation cohorts, respectively. Kaplan–Meier curves confirmed effective patient stratification (*p* < 0.001) in both test and validation cohorts. A high Radscore, rough intestinal outer edge, and advanced age were identified as key prognostic risk factors using the SHAP.

**Conclusion:**

The combined model effectively stratified patients with stage II CRC into different prognostic risk groups, aiding clinical decision-making.

**Critical relevance statement:**

Integrating CT images with clinicopathological information can facilitate the identification of patients with stage II CRC who are most likely to benefit from adjuvant chemotherapy.

**Key Points:**

- The effectiveness of adjuvant chemotherapy for stage II colorectal cancer remains debated.
- A combined model successfully identified high-risk stage II colorectal cancer patients.
- Shapley additive explanations enhance the interpretability of the model’s predictions.

**Graphical Abstract:**

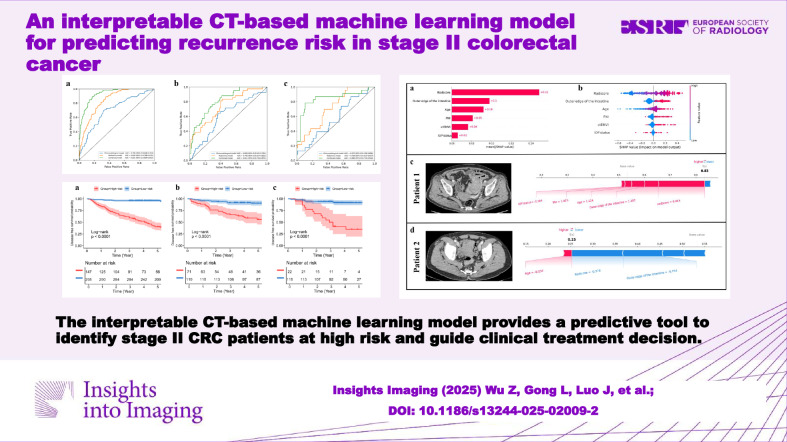

## Introduction

Colorectal cancer (CRC) is the most common gastrointestinal cancer and ranks third in terms of morbidity and mortality among malignant tumors [1]. Stage II colorectal cancer is an early-stage tumor that has not metastasized to lymph nodes or distant organs [2]. Surgical resection is the primary treatment for locoregional colorectal cancer [3], but 20–25% of patients experience fatal disease recurrence after surgery [4]. Adjuvant chemotherapy (ACT) helps eliminate micrometastases and surgical remnants, thereby reducing the risk of recurrence. However, considerable debate remains regarding the use of adjuvant chemotherapy in patients with stage II colorectal cancer [5].

According to current clinical guidelines, ACT is usually recommended for patients with stage II CRC who have one or more of the following high-risk factors: T4 lesions, poorly differentiated tumors, intestinal obstruction or perforation (IOP) status, lymphovascular invasion (LVI), perineural invasion (PNI), positive postoperative margins, and a small number (< 12) of lymph nodes examined after surgery [6]. However, these clinicopathological risk factors do not accurately identify high-risk patients, leading to recurrence in 13% of low-risk patients [5, 7]. Therefore, there is an urgent need for more accurate predictors to improve the diagnostic performance of clinicopathological risk factors in stratifying patients with high-risk stage II cancer.

Radiomics, a technique for extracting high-throughput quantitative features from routine medical images, has the potential to characterize complex tumor phenotypes. The integration of machine learning has enabled radiomics to achieve greater accuracy and efficiency in cancer diagnosis, prognosis, and treatment response assessment [8, 9]. Although several studies have successfully applied radiomics to enhance the accuracy of prognostic evaluation of stage II CRC [10–12], exploration of imaging features remains scarce. Additionally, the lack of multicenter validation and interpretability of machine learning models limits their use in clinical practice.

This study aimed to develop a reliable machine learning tool that incorporates clinicopathological factors, imaging signs, and tumor radiomics information to identify high-risk stage II CRC patients, thus facilitating practical clinical decision-making.

## Materials and methods

### Patients

This retrospective study was approved by the Institutional Review Boards (ethics approval number: GDREC2020011H) and the requirement for informed consent was waived because of the retrospective nature of the study. This study consecutively included patients from Nanfang Hospital of Southern Medical University (Hospital 1), Guangdong Provincial People’s Hospital (Hospital 2), and Guangxi Medical University Cancer Hospital (Hospital 3).

**Fig. 1:**
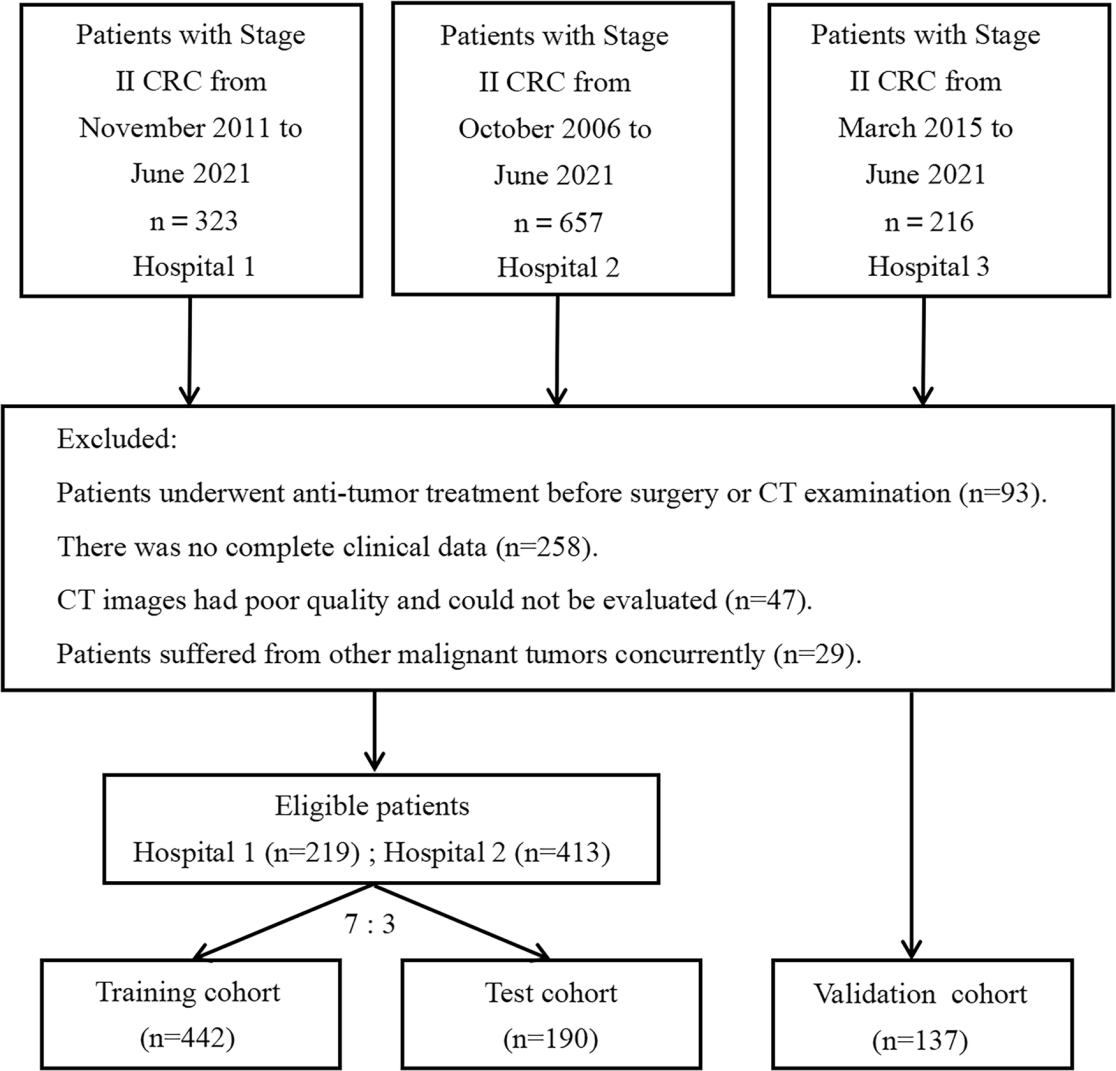
Flowchart of inclusion and exclusion criteria for eligible patients in the study

We retrospectively recruited 769 patients with pathologically confirmed stage II CRC who underwent radical resection between October 2006 and June 2021 at three hospitals. The inclusion and exclusion processes are illustrated in Fig. 1. Finally, 632 individuals recruited from hospitals 1 and 2 were randomly assigned to the training cohort (*n* = 442) or test cohort (*n* = 190) at a ratio of 7:3. In addition, 137 patients from hospital 3 were included in the validation cohort.

### Clinicopathological data and follow-up

**Fig. 2:**
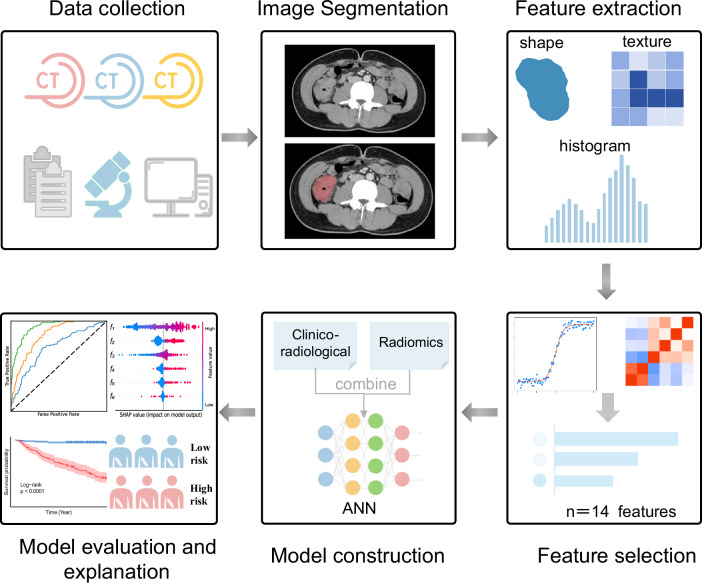
The workflow of this study. ANN, artificial neural network

Patient clinical data, including age, sex, tumor location, T-stage, number of lymph nodes examined, LVI, PNI, IOP status, and adjuvant chemotherapy, were recorded from the medical record archives. Routine follow-up visits were performed every 3–6 months during the first 2–3 years and every 6–12 months thereafter. The last tracking date was June 30, 2024. Disease-free survival (DFS) was defined as the duration from the date of surgery to the first occurrence of recurrence, metastasis, death from any cause, or last follow-up. Patients with DFS events within 3 years were classified into the recurrence group, whereas those without DFS events were classified into the non-recurrence group. Tumor staging was performed according to the 8th edition of the American Joint Committee on Cancer (AJCC) staging system [4]. Figure 2 shows the study design and workflow.

### CT acquisition and imaging features evaluation

The CT scanners and scanning parameters for each institution are listed in Supplementary Table 1. Two radiologists with 5 (Z.Q.W.) and 6 (L.Y.G.) years of experience in abdominal diagnosis were invited to independently assess the CT imaging features; they were blinded to the clinical and histopathological information. Any disagreements were resolved through discussion with a third radiologist (G.W.) with 33 years of experience in abdominal diagnosis until a consensus was reached. All the results were corroborated by a third radiologist. The following CT imaging features were evaluated: (1) tumor morphology; (2) tumor margin; (3) outer edge of the intestine; (4) enhancement degree; (5) enhancement pattern; (6) the hypoattenuation-within-tumor ratio; (7) peritumoral adipose tissue; (8) CT-detected extramural venous invasion(ctEMVI); (9) length; (10) thickness. The evaluation criteria for these CT features are detailed in Supplementary Table 2.

### Image pre-processing and tumor segmentation

All original CT images were appropriately preprocessed to minimize batch effects from different hospitals and scanners. To standardize the voxel spacing, the images were resampled to 1 × 1 × 1 mm^3^ (x, y, z) using a linear interpolation algorithm. A 25 HU bin width was then set to discretize the voxel intensity and reduce noise [13–15].

Axial images of the venous phase were imported into the in-house software DeepCRC [16] for automatic tumor segmentation. Subsequently, two radiologists with 5 years (Z.Q.W.) and 6 years (L.Y.G.) of experience in diagnosing abdominal lesions jointly reviewed and corrected the segmentation results layer-by-layer using ITK-SNAP software (version 3.6.0). Corrected results from both radiologists were used as the final volumes of interest (VOIs). In cases of disagreement, a third radiologist (G.W.) with 33 years’ experience in abdominal diagnosis was consulted to reach a consensus through discussion.

### Radiomics feature extraction and selection

The radiomic features of the tumor VOI, including shape, first-order, texture, wavelet, and square transform features, were automatically extracted using the PyRadiomics package [17] (version 3.8.8). The radiomics feature values were normalized using the z-score method. To mitigate model bias toward the majority class, an upsampling method was applied to balance the ratio between positive and negative samples. Initially, the Mann–Whitney U test was performed to select features related to prognosis (*p* < 0.05). The Spearman’s rank correlation test was then used to exclude redundant features (correlation coefficient value ≥ 0.5). Finally, the elastic net algorithm was used to identify the most predictive features for constructing a radiomics model.

### Construction of radiomics model

Five different ML algorithms–logistic regression (LR), decision tree (DT), random forest (RF), eXtreme Gradient Boosting (XGBoost), and artificial neural network (ANN)–were used to construct a radiomics model. To prevent the overfitting of the model, a grid search and 5-fold cross-validation method were used to validate the algorithm. For further analysis, the model with the best performance was selected. The output risk probability was regarded as the Radscore.

### Construction of clinicoradiological model

Univariate logistic regression analysis was conducted on both clinical and radiological characteristics to identify significant factors (*p* < 0.05). Subsequently, multivariate logistic regression was performed to determine the independent risk factors associated with recurrence, leading to the construction of a clinicoradiological model.

### Construction of combined model

The Radscore, calculated using the highest-performing radiomics model, was used to construct a combined model. This model was developed using the ANN algorithm, which integrates the Radscore with significant clinicopathological risk features.

### Interpretability analysis of predictive model

In this study, the Shapley additive explanations (SHAP) algorithm was used to interpret the best-performing ML model and address the “black box” challenge [18]. The SHAP algorithm reveals the contribution of each feature to the model’s diagnosis, offering both global and local interpretability along with visual representation [19]. To facilitate interpretation, a SHAP summary plot was used to rank the influential features based on their impact. SHAP force plots were generated to explain the individual diagnoses in several representative cases, illustrating how the ML model works.

### Prognosis stratification analysis of the optimal model

The Kaplan–Meier method was used to analyze survival outcomes in the low-and high-risk groups stratified by the combined model and to create corresponding survival curves. Additionally, a log-rank test was conducted to evaluate the statistical significance of the differences between groups.

### Statistical analysis

Continuous variables are reported as medians and interquartile ranges, and categorical variables are reported as numbers and percentages. Univariate and multivariate logistic regression analyses were used to identify risk factors with odds ratios (OR) and 95% confidence intervals (CI). The performance of each model was evaluated using the receiver operating characteristic (ROC) curve, area under the curve (AUC), accuracy, sensitivity, and specificity. The DeLong test was used to compare AUC differences. Calibration curves were plotted to evaluate the calibration of the model, and decision curve analysis (DCA) was performed to estimate the clinical utility of the prediction model. The incremental differences between the single and combined models were compared using the net reclassification index (NRI) and the integrated discrimination improvement (IDI). The SHAP analysis was implemented using the SHAP package (https://github.com/slundberg/shap). Statistical significance was set at *p* < 0.05. Statistical analyses were performed using R software (version 4.3.1).

## Results

### Patient characteristics

The clinicopathological characteristics of patients in the three cohorts are presented in Table 1. Of the 769 patients included in the study, 476 were men and 293 were women. The recurrence rates within 3 years in the three cohorts were 21.9%, 22.1%, and 16.8%, respectively. In the three cohorts, 60% of the patients received chemotherapy after surgery (training cohort: 62.0%; test cohort: 61.1%; validation cohort: 56.2%).

**Table 1:** Characteristics of patients in three cohorts

Characteristics	Training cohort (*n* = 442)	Test cohort (*n* = 190)	Validation cohort (*n* = 137)
3-year recurrence, *n* (%)			
No	345 (78.1%)	148 (77.9%)	114 (83.2%)
Yes	97 (21.9%)	42 (22.1%)	23 (16.8%)
Age (years), median [IQR]	61.0 [50.8, 71.0]	60.0 [51.0, 67.0]	63.0 [51.0, 70.0]
Sex, *n* (%)			
Male	272 (61.5%)	119 (62.6%)	85 (62.0%)
Female	170 (38.5%)	71 (37.4%)	53 (38.0%)
Location, *n* (%)			
Right colon	186 (42.1%)	84 (44.2%)	43 (31.4%)
Left colon	116 (26.2%)	41 (21.6%)	69 (50.4%)
Rectum	140 (31.7%)	65 (34.2%)	25 (18.2%)
Chemotherapy, *n* (%)			
No	168 (38.0%)	74 (38.9%)	60 (43.8%)
Yes	274 (62.0%)	116 (61.1%)	77 (56.2%)
pT, *n* (%)			
T3	365 (82.6%)	157 (82.6%)	75 (54.8%)
T4	77 (17.4%)	33 (17.4%)	62 (45.2%)
Number of lymph nodes examined, *n* (%)			
< 12	97 (21.9%)	45 (23.7%)	30 (21.9%)
≥ 12	345 (78.1%)	145 (76.3%)	107 (78.1%)
LVI, *n* (%)			
Negative	397 (89.8%)	174 (91.6%)	105 (76.6%)
Positive	45 (10.2%)	16 (8.4%)	32 (23.4%)
PNI, *n* (%)			
Negative	366 (82.8%)	160 (84.2%)	66 (48.2%)
Positive	76 (17.2%)	30 (15.8%)	71 (51.8%)
IOP status, *n* (%)			
Negative	416 (94.1%)	180 (94.7%)	115 (83.9%)
Positive	26 (5.9%)	10 (5.3%)	22 (16.1%)
Length (mm), median [IQR]	52.0 [40.0, 66.0]	50.0 [36.0, 63.0]	56.0 [45.5, 68.0]
Thickness (mm), median [IQR]	14.0 [11.0, 18.0]	14.0 [11.0, 18.0]	13.8 [10.0, 16.0]
Morphology, *n* (%)			
Infltrating or ulcerative type	416 (94.1%)	156 (82.1%)	101 (73.7%)
Mass type	26 (5.9%)	34 (17.9%)	36 (26.3%)
Margin, *n* (%)			
Well-defined	157 (35.5%)	66 (34.7%)	37 (27.0%)
Ill-defined	285 (64.5%)	124 (65.3%)	100 (73.0%)
Outer edge of the intestine, *n* (%)			
Smooth	313 (70.8%)	141 (74.2%)	97 (70.8%)
Rough	129 (29.2%)	49 (25.8%)	40 (29.2%)
Enhancement degree, *n* (%)			
Hyper/isoenhancement	424 (95.9%)	179 (94.2%)	131 (95.6%)
Hypoenhancement	18 (4.1%)	11 (5.8%)	6 (4.4%)
Enhancement pattern, *n* (%)			
Heterogeneous	160 (36.2%)	62 (32.6%)	47 (34.3%)
Homogeneous	282 (63.8%)	128 (67.4%)	90 (65.7%)
The hypoattenuation-within-tumor ratio, *n* (%)			
< 1/3	325 (73.5%)	140 (73.7%)	101 (73.7%)
1/3–2/3	72 (16.3%)	29 (15.2%)	20 (14.6%)
> 2/3	45 (10.2%)	21 (11.1%)	16 (11.7%)
Peritumoral adipose tissue, *n* (%)			
Clean	372 (84.2%)	162 (85.3%)	117 (85.4%)
Dirty	70 (15.8%)	28 (14.7%)	20 (14.6%)
ctEMVI, *n* (%)			
Negative	372 (84.2%)	156 (82.1%)	112 (81.6%)
Positive	70 (15.8%)	34 (17.9%)	25 (18.4%)
Futime (month), median [IQR]	70.5 [43.0, 97.0]	76.0 [46.8, 100.3]	42.0 [35.5, 57.0]

LVI-positive: the presence of cancer cells in the lumen of the blood vessels and/or the lumen of the lymphatic vessels, PNI-positive: at least 33% of the perinerve is surrounded by cancer cells (not invading the nerve sheath), IOP status-positive: preoperative intestinal obstruction or localized perforation

*ctEMVI* CT-detected extramural venous invasion, *IOP* intestinal obstruction or perforation, *LVI* lymphovascular invasion, *PNI* perineural invasion

### Radiomics model construction and performance

First, 944 radiomics features derived from the tumors were selected for further study. After a series of feature selections, the 14 most valuable radiomics features were selected to develop the radiomics model using the ML algorithm (Supplementary Fig. 1 and Supplementary Table 3). As shown in Table 2, the ANN model based on radiomics exhibited the best performance, producing the highest AUC values of 0.839 (95% CI: 0.798–0.875), 0.742 (95% CI: 0.654–0.821) and 0.720 (95% CI: 0.610–0.817) in the training, test, and validation cohort, respectively. The architecture of the ANN is presented in Supplementary Text 1 and Fig. 2.

**Table 2:** Performance of different radiomics models

Cohort	Model	AUC (95% CI)	Accuracy	Sensitivity	Specificity
Training cohort	LR	0.697 (0.660–0.734)	0.686	0.876	0.472
	RF	0.800 (0.768–0.828)	0.739	0.773	0.701
	Xgboost	0.758 (0.722–0.791)	0.711	0.763	0.652
	DT	0.776 (0.743–0.809)	0.693	0.546	0.858
	ANN	0.839 (0.798–0.875)	0.705	0.891	0.646
Test cohort	LR	0.602 (0.513–0.703)	0.479	0.714	0.412
	RF	0.627 (0.523–0.725)	0.595	0.548	0.608
	Xgboost	0.670 (0.577–0.767)	0.595	0.690	0.568
	DT	0.658 (0.566–0.744)	0.674	0.476	0.730
	ANN	0.742 (0.654–0.821)	0.647	0.619	0.655
Validation cohort	LR	0.632 (0.494–0.766)	0.759	0.348	0.842
	RF	0.611 (0.477–0.734)	0.796	0.087	0.939
	Xgboost	0.662 (0.518–0.788)	0.613	0.565	0.623
	DT	0.599 (0.483–0.715)	0.584	0.652	0.570
	ANN	0.720 (0.610–0.817)	0.839	0.217	0.965

*ANN* artificial neural network, *AUC* area under the curve, *DT* decision tree, *LR* logistic regression, *RF* random forest, *Xgboost* eXtreme Gradient Boosting

### Clinicoradiological model construction and performance

**Fig. 3:**
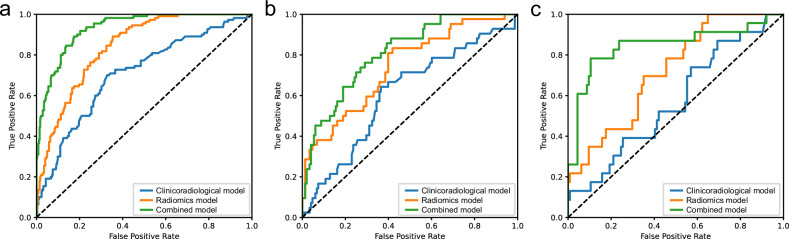
ROC curve of the radiomics, clinicoradiological, and combined model in the training cohort (**a**), the test cohort (**b**), and the validation cohort (**c**)

The training cohort was subjected to both univariate and multivariate logistic analyses based on radiological and clinical characteristics. The results are presented in Table 3. The results showed that age, PNI, IOP status, the outer edge of the intestine, and ctEMVI were independent predictors of recurrence and were used to construct a clinicoradiological model. The model predicted an AUC of 0.706 (95% CI: 0.648–0.763) for recurrence in the training cohort, 0.608 (95% CI: 0.503–0.704) in the test cohort, and 0.572 (95% CI: 0.438–0.696) in the validation cohort (Table 4, Fig. 3).

**Table 3:** Results of logistic regression in the training cohort

Characteristic	Univariate analysis		Multivariate analysis	
	OR (95% CI)	*p*-value	OR (95% CI)	*p*-value
Age	1.03 (1.02–1.05)	< 0.001	1.03 (1.02–1.05)	< 0.001
Sex	1.02 (0.75–1.40)	0.89	-	-
Location	0.92 (0.77–1.10)	0.38	-	-
Chemotherapy	0.82 (0.60–1.12)	0.21	-	-
pT	0.87 (0.58–1.31)	0.51	-	-
Number of lymph nodes examined	0.70 (0.49–1.01)	0.06	-	-
LVI	1.31 (0.81–2.13)	0.27	-	-
PNI	2.35 (1.58–3.48)	< 0.001	2.41 (1.59–3.66)	< 0.001
IOP status	3.09 (1.62–5.89)	< 0.001	3.17 (1.59–6.34)	0.001
Length	0.99 (0.99–1.00)	0.11	-	-
Thickness	1.00 (0.98–1.02)	0.99	-	-
Morphology	0.70 (0.46–1.08)	0.11	-	-
Margin	1.50 (1.08–2.07)	0.02	1.12 (0.79–1.59)	0.52
Outer edge of the intestine	1.91 (1.37–2.67)	< 0.001	1.60 (1.05–2.44)	0.03
Enhancement degree	0.78 (0.36–1.72)	0.55	-	-
Enhancement pattern	1.08 (0.79–1.47)	0.63		
The hypoattenuation-within-tumor ratio	1.10 (0.87–1.38)	0.42	-	-
Peritumoral adipose tissue	1.09 (0.72–1.66)	0.68	-	-
ctEMVI	2.05 (1.36–3.09)	< 0.001	1.88 (1.13–3.18)	0.02

*ctEMVI* CT-detected extramural venous invasion, *IOP* intestinal obstruction or perforation, *LVI* lymphovascular invasion, *OR* odds ratio, *PNI* perineural invasion

**Table 4:** Clinicoradiological, radiomics, and combined model performance

Cohort	Model	AUC (95% CI)	Accuracy	Sensitivity	Specificity
Training cohort	Clinicoradiological model	0.706 (0.648–0.763)	0.679	0.700	0.672
	Radiomics model	0.839 (0.798–0.875)	0.705	0.891	0.646
	Combined model	0.932 (0.909–0.953)	0.842	0.891	0.826
Test cohort	Clinicoradiological model	0.608 (0.503–0.704)	0.632	0.524	0.662
	Radiomics model	0.742 (0.654–0.821)	0.647	0.619	0.655
	Combined model	0.811 (0.738–0.874)	0.732	0.738	0.730
Validation cohort	Clinicoradiological model	0.572 (0.438–0.696)	0.482	0.739	0.430
	Radiomics model	0.720 (0.610–0.817)	0.839	0.217	0.965
	Combined model	0.846 (0.730–0.944)	0.876	0.609	0.930

*AUC* area under the curve, *CI* confidence interval

### Combined model construction and performance

**Fig. 4:**
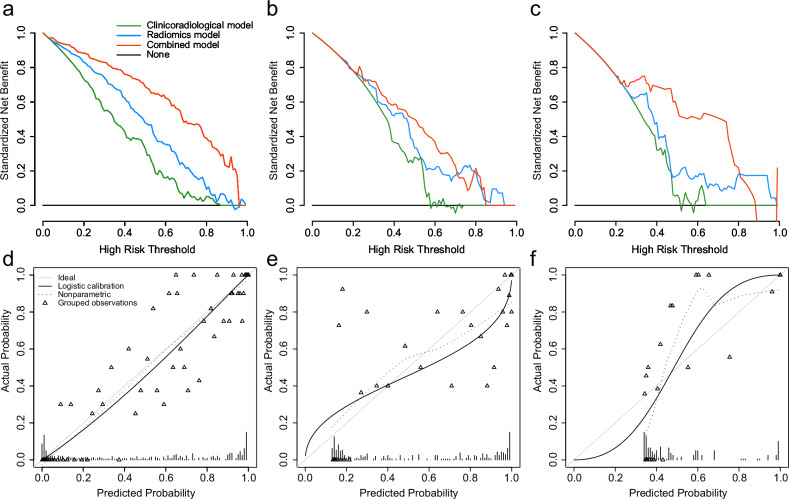
The decision curve analysis of radiomics, clinicoradiological, and combined model in the training cohort (**a**), the test cohort (**b**), and the validation cohort (**c**); the calibration curve of the combined model in the training cohort (**d**), the test cohort (**e**), and the validation cohort (**f**)

Table 4 and Fig. 3 show the AUC, accuracy, sensitivity, and specificity of the radiomics, clinicoradiological, and combined models in the training, test, and validation cohorts, respectively. Among all the models, the combined model used ANN algorithm was the best predictor of recurrence risk (AUCs of 0.932, 0.811, and 0.846 in the training, test, and validation cohorts, respectively), outperforming the radiomics model (DeLong’s test, *p* < 0.05 in the training, test cohort and *p* = 0.053 in the validation cohort); it also outperformed the clinicoradiological model (DeLong’s test, all *p* < 0.01). DCA indicated that the combined model had a higher net benefit in predicting recurrence than the clinicoradiological and radiomics models in the entire cohorts (Fig. 4a–c). The calibration curves of the prediction model showed good agreement between the model-predicted and actual probabilities (Fig. 4d–f). Furthermore, compared with the clinicoradiological model that incorporated only clinical and radiological risk predictors, the use of radiomics significantly improved the prediction performance of the combined model for recurrence in terms of NRI and IDI (Supplementary Table 4).

### Interpretability analysis of ANN-combined model

**Fig. 5:**
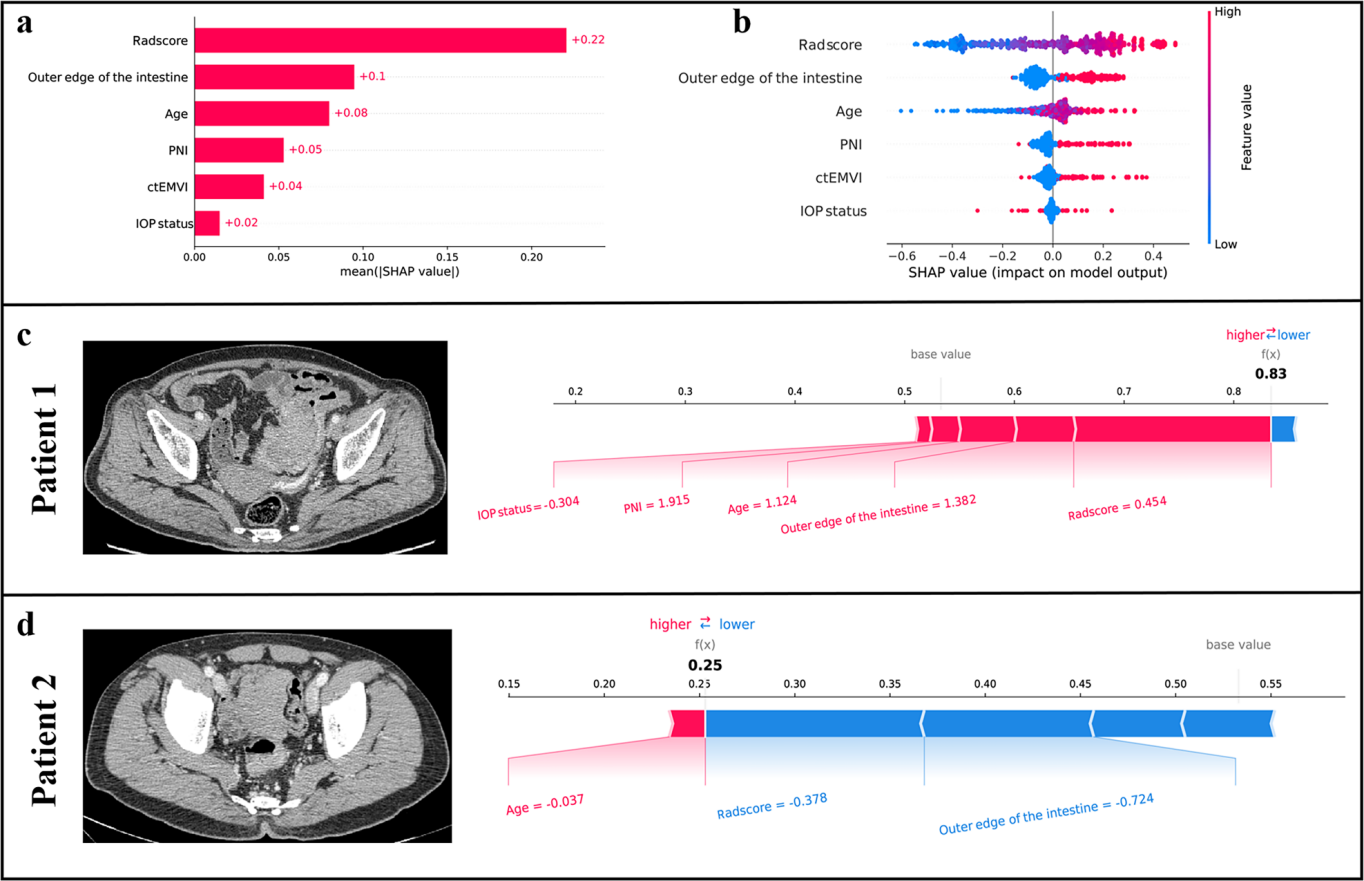
The SHAP feature importance plots. **a** The plot ranked the importance of each feature for the global prediction result. **b** The SHAP beeswarm plot. The plot showed the distribution of the effect of each feature on the model output. **c** The SHAP explanation force plots for patient 1 were classified as recurrence. **d** The SHAP explanation force plots for patient 2 were classified as non-recurrence

The SHAP feature importance plots exhibited six prominent features: Radscore, age, and outer edge of the intestine in the ANN-combined model, followed by ctEMVI, PNI, and IOP status (Fig. 5a). The plot shows the distribution of the Shapley values, ranging from high to low, representing the impact of each feature on the model’s output. In the SHAP beeswarm plot (Fig. 5b), we observed an association between a feature’s SHAP value and its impact on the prediction. A higher SHAP value indicated a greater probability of recurrence. Figure 5c, d displays the SHAP explanatory force plots for two representative cases, which demonstrate how the features of the model influence its output. In a 77-year-old patient (patient 1) with a high Radscore (0.454), intestinal obstruction, PNI-positive, and rough outer edge of the intestine, the model correctly predicted recurrence. A 61-year-old patient (patient 2) had a lower Radscore (−0.378) and smooth outer edge of the intestine was correctly predicted by the model to be non-recurrence.

### Prognosis stratification ability of the optimal model

**Fig. 6:**
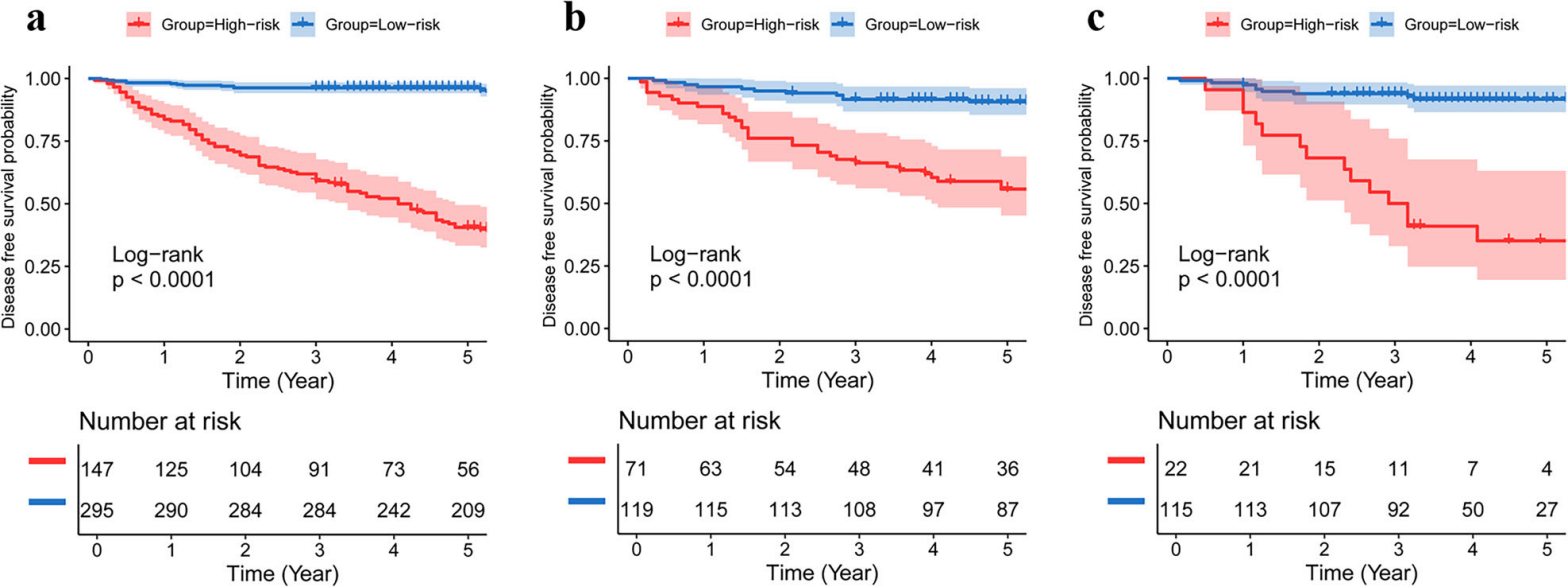
Kaplan–Meier curve of disease-free survival based on the combined model in the training (**a**), test (**b**), and validation (**c**) cohorts

All patients in the three cohorts were classified into low- and high-risk groups based on the optimal cutoff value of the combined model, as determined by the maximum Youden index (0.464) in the training cohort. Survival comparisons between patients at different risk levels suggested a much worse prognosis in patients with high-risk than in patients with low-risk in the training cohort (*p* < 0.0001, HR = 7.364, 95% CI: 5.342–10.151; Fig. 6a), test cohort (*p* < 0.0001, HR = 2.744, 95% CI: 1.818–4.144; Fig. 6b) and validation cohort (*p* < 0.0001, HR = 8.630, 95% CI: 3.976–18.734; Fig. 6c). The combined model could successfully stratify patients with stage II CRC and identify those with a poor prognosis.

## Discussion

In this study, we developed and validated a prognostic model that integrates tumor radiomic information and clinicoradiological features. By applying machine learning, we developed an optimal prognostic model and successfully stratified patients with stage II CRC into significantly different prognostic risk groups. The SHAP method was used to visualize the entire model prediction process, providing important information for clinical decision-making.

In the current clinical practice, the risk of recurrence is primarily stratified based on clinicopathological factors. Hoshino et al established a predictive nomogram for recurrence in stage II CRC, with a C-index of 0.64, based only on several clinicopathological characteristics [20]. However, these clinicopathological risk factors are not sufficiently accurate to identify patients at high risk of recurrence, and more information should be utilized to enhance the comprehensiveness of clinical assessment. To assess the biological behavior of the tumors more comprehensively, we evaluated their imaging features. In this study, among the five clinicoradiological factors, the outer edge of the intestine contributed the most to predicting recurrence risk. The outer edge of the intestine tends to reflect the aggressiveness and growth pattern of the tumor, with rough or irregular walls indicating deeper tissue invasion, which increases the risk of recurrence. ctEMVI positivity indicates that the tumor has invaded the blood vessels beyond the intestinal wall, providing a pathway for tumor cell progression and migration, which affects tumor prognosis [21, 22]. Previous studies have also demonstrated that age, PNI, and IOP status are clinicopathological factors affecting recurrence risk, which is consistent with our research results [10, 23]. The clinicoradiological model demonstrated modest performance, with AUCs of 0.706, 0.608, and 0.572 for the training, test, and validation cohorts, respectively. The unsatisfactory performance of the clinicoradiological model indicates the need to develop a better predictive model.

Radiomics can obtain deep-level information that cannot be recognized by the naked eye, providing a non-invasive approach for tumor diagnosis, staging, and prognosis prediction [24]. In this study, 14 optimal features associated with tumor recurrence were selected for modeling. Low gray-level zone emphasis, flatness, and small area low gray-level emphasis were identified as the top three radiomic features contributing to the Radscore. Our results showed that the values of two texture features, low gray-level zone emphasis and small area low gray-level emphasis, were higher in patients with recurrence than in those without recurrence. This finding suggests that greater tumor heterogeneity [24] and extensive necrosis are associated with a poorer prognosis, consistent with previous reports [25]. Additionally, our results indicate that tumors with higher flatness are more prone to recurrence. A higher flatness represents a non-flat, sphere-like tumor [26], which may be linked to their expansive growth, making these tumors more likely to invade adjacent tissues and metastasize. Fan et al reported that a CT radiomics nomogram combined with clinical factors performed well in predicting recurrence risk in stage II CRC, with an AUC of 0.906 in the validation dataset [10]. In another study by Cao et al, deep learning models based on radiomics achieved an AUC of 0.76 in the testing cohort for predicting DFS in stage II CRC [12]. However, neither study included patients from external centers to validate the generalizability and stability of the model. This multicenter study involved three independent medical institutions with a total sample size of 769. Moreover, this study incorporated imaging features and constructed a combined model that provided additional information (all *p* < 0.001 in the validation cohort, the NRI test, and the IDI test). We further explored the prognostic information of the combined model, and the preliminary results of this study indicated that the combined model could predict tumor recurrence stratification (HR: 2.744–8.630; all *p* < 0.001). For patients in the high-risk group, more frequent follow-ups and aggressive individualized treatments should be implemented.

Our findings indicate that models relying on traditional machine learning algorithms such as logistic regression and random forest exhibit limited performance, likely because of their inefficiency in processing complex and nonlinear data. Therefore, we integrate the ANN algorithm into our proposed methodology. The ANN algorithm effectively handles complex nonlinear relationships by simulating biological neural networks [27–29]. It has been shown to outperform traditional discriminant analysis and is emerging as a new standard for disease risk prediction [30]. To help clinicians better understand the feature contributions and reasoning process of the model, we visualized these processes using the SHAP algorithm [31]. To enhance the local interpretability, we utilized SHAP force plots to illustrate the impact of individual features on the prediction process for each instance. Moreover, the interpretation informs clinicians about the elements of the ML model that may add value, thereby guiding the model’s refinement as a clinical decision-making aid [32].

Our study has some limitations. First, as a retrospective study, it may have led to an information selection bias. Second, we focused only on CT images of the portal venous phase to develop a radiomic signature. Further research is needed to determine whether noncontrast and arterial phase images can provide additional information for estimating the prognosis of patients with stage II colorectal cancer. Third, although our model successfully stratified patients into high- and low-risk groups in terms of prognosis, demonstrating its potential value in guiding clinical treatment decisions, it lacks validation in prospective cohorts to assess the actual benefits of adjuvant chemotherapy in the high-risk group.

## Conclusion

We developed and validated a robust combined model that integrates CT radiomics and clinicoradiological features using machine learning to predict recurrence and stratify patients with stage II colorectal cancer. The model incorporates interpretable analysis using the SHAP algorithm and is expected to provide further guidance for clinical practice.

## Supplementary information

ELECTRONIC SUPPLEMENTARY MATERIAL

## Data Availability

The datasets generated and analyzed during the current study are not publicly available but are available from the corresponding author on reasonable request.

## Abbreviations

ACT: Adjuvant chemotherapy
AJCC: American Joint Committee on Cancer
ANN: Artificial neural network
AUC: Area under the curve
CI: Confidence interval
CRC: Colorectal cancer
ctEMVI: CT-detected extramural venous invasion
DFS: Disease-free survival
DT: Decision tree
IDI: Integrated discrimination improvement
IOP: Intestinal obstruction or perforation
LR: Logistic regression
LVI: Lymphovascular invasion
ML: Machine learning
NRI: Net reclassification index
OR: Odds ratio
PNI: Perineural invasion
RF: Random forest
ROC: Receiver operating characteristic
SHAP: Shapley additive explanations
VOI: Volume of interest
XGBoost: eXtreme Gradient Boosting
