# Supplementary material for: An interpretable CT-based machine learning model for predicting recurrence risk in stage II colorectal cancer
Source: Insights Imaging. 2025 Jul 31;16:162. doi: 10.1186/s13244-025-02009-2 (PMC12314294; doi:10.1186/s13244-025-02009-2)
Supplement: Supplementary file 1 — ELECTRONIC SUPPLEMENTARY MATERIAL [file 13244_2025_2009_MOESM1_ESM.pdf]

# An interpretable CT-based machine learning model for predicting recurrence risk in stage II colorectal cancer

## ELECTRONIC SUPPLEMENTARY MATERIAL

**Supplementary Text**

**Supplementary Text 1.** Artificial neural network model

Selected radiomics features with and without important clinicoradiological features serve as the inputs to the neural network to construct radiomics model or combined model, respectively, while tumor recurrence is the output of the neural network. Sigmoid activation functions were employed in the input-hidden layer and hidden-output layer. The Sigmoid activation functions can be described as follows:

$$\begin{cases} X = \sum_{i=1}^{n} W_i x_i + B \\ Sigmoid(X) = \dfrac{1}{1 + e^{-X}} \end{cases}$$

Where $i$ represent different input features, ranging from 1 to $n$ (n represents the total number of input features). $X$ is the linearly weighted sum of the input features $x$, and $W$ is the feature weight, $B$ is the constant term of activation function. $Sigmoid(X)$ are the formula of sigmoid activation.

**Supplementary Table**

**Supplementary Table 1.** CT Scanning Parameters

| Hospital | Scanner | Tube Voltage (kV) | Tube Current (mAs) | Detector or Collimation (mm) | FOV (mm) | Slice Interval (mm) | Slice Thickness (mm) |
|---|---|---|---|---|---|---|---|
| I | Philips Brilliance iCT | 120 | 159 | 256 | 350x350 | 5 | 5 |
| | Somatom Definition Flash | 120 | 159 | 128 | 350x350 | 5 | 5 |
| II | Philips Brilliance iCT | 120 | 150 | 256 | 350x350 | 5 | 5 |
| | GE LightSpeed VCT | 120 | 150 | 64 | 350x350 | 5 | 5 |
| III | GE Discovery CT750 HD | 120 | 220 | 128 | 360x360 | 5 | 5 |
| | somatom sensation 64 | 120 | 210 | 64 | 391x391 | 5 | 5 |

**Supplementary Table 2.** CT-based assessment of tumor-related features

| Features | Assessment | Classification |
| --- | --- | --- |
| Tumor morphology | Determine if the tumor is located in the lumen or extends along the intestinal wall | Mass type, infltrating or ulcerative type |
| Tumor margin | Tumor with a gradual transition or a sharp transition from the adjacent normal bowel | Ill-defined margin, well-defined margin |
| Outer edge of the intestine | Evaluated the smoothness of wall | Smooth, rough |
| Enhancement degree | Defined as hyper/isoenhancement or hypoenhancement compared to the adjacent colon | Hyper/isoenhancement or hypoenhancement |
| Enhancement pattern | Uniform or heterogeneous enhancement across a lesion or tissue | Homogeneous, heterogeneous |
| The hypoattenuation-within-tumor ratio | The proportion of poor or no enhancement within the tumor | < 1/3, 1/3-2/3, > 2/3 |
| Peritumoral adipose tissue | Absence or presence of strands and/or nodules in peritumoral adipose tissue | Clean，dirty |
| ctEMVI | Absence of vessels near the tumor or vascular-like enhancement in vessels beyond the bowel wall indicates ctEMVI-<br>Soft tissue density in the lumen beyond the bowel wall, an enlarged lumen, and/or an irregular border is ctEMVI+ [1] | ctEMVI-, ctEMVI+ |
| Length | measured longitudinally along the course of the colorectal | |
| Thickness | measured perpendicular to colorectal lumen | |

ctEMVI, CT-detected extramural venous invasion

**Supplementary Table 3.** Detailed of selected radiomics features

| Number | Filter | Type | Name |
|---|---|---|---|
| 1 | original | shape | Flatness |
| 2 | original | gldm | DependenceVariance |
| 3 | square | gldm | DependenceVariance |
| 4 | wavelet.LHL | firstorder | Median |
| 5 | wavelet.LHH | firstorder | Median |
| 6 | wavelet.LHH | firstorder | Skewness |
| 7 | wavelet.HLL | glcm | ClusterShade |
| 8 | wavelet.HLL | glcm | MaximumProbability |
| 9 | wavelet.HLL | glszm | SmallAreaLowGrayLevelEmphasis |
| 10 | wavelet.HHL | glszm | LowGrayLevelZoneEmphasis |
| 11 | wavelet.HHH | glrlm | GrayLevelNonUniformityNormalized |
| 12 | wavelet.HHH | glszm | LowGrayLevelZoneEmphasis |
| 13 | wavelet.LLL | firstorder | Skewness |
| 14 | wavelet.LLL | glcm | MaximumProbability |

**Supplementary Table 4.** NRI and IDI of the combined model versus clinicoradiological model and radiomics model

| Cohort | Model | NRI | 95％CI | *P* value | IDI | 95％CI | *P* value |
|---|---|---|---|---|---|---|---|
| Training cohort | clinicoradiological model | 1.417 | 1.274-1.559 | ＜0.001 | 0.407 | 0.364-0.449 | ＜0.001 |
| | radiomics model | 0.935 | 0.742-1.129 | ＜0.001 | 0.234 | 0.182-0.287 | ＜0.001 |
| Test cohort | clinicoradiological model | 0.861 | 0.552- 1.172 | ＜0.001 | 0.180 | 0.121-0.239 | ＜0.001 |
| | radiomics model | 0.299 | -0.039-0.636 | 0.083 | 0.043 | -0.004 - 0.090 | 0.072 |
| Validation cohort | clinicoradiological model | 1.286 | 0.901-1.671 | ＜0.001 | 0.268 | 0.146 - 0.389 | ＜0.001 |
| | radiomics model | 0.864 | 0.467-1.261 | ＜0.001 | 0.134 | 0.063- 0.205 | ＜0.001 |

The *P* value was calculated by comparing with the combined model. NRI net reclassification index, IDI integrated discrimination improvement

## Supplementary Figures

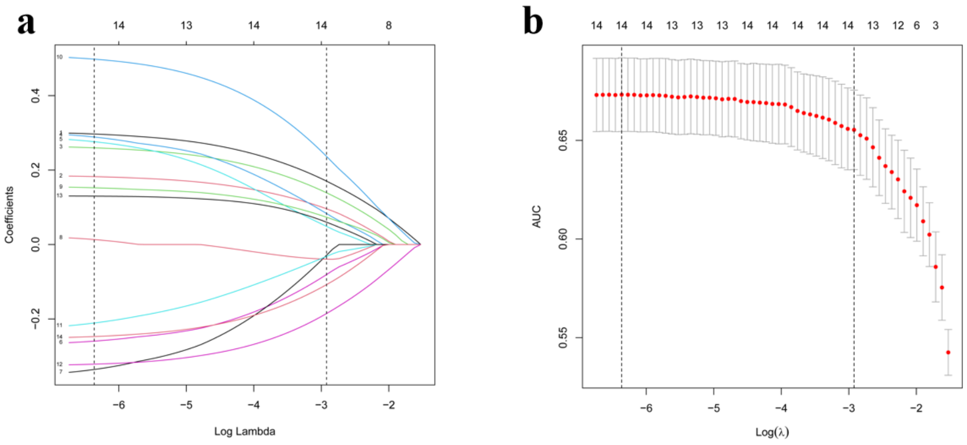


**Supplementary Figure 1.** Feature selection with elastic net algorithm. (a) Elastic net coefficient analysis of the 14 radiomics features. (b) Tuning parameter λ in the elastic net model. The parameters were selected under the minimum criteria.

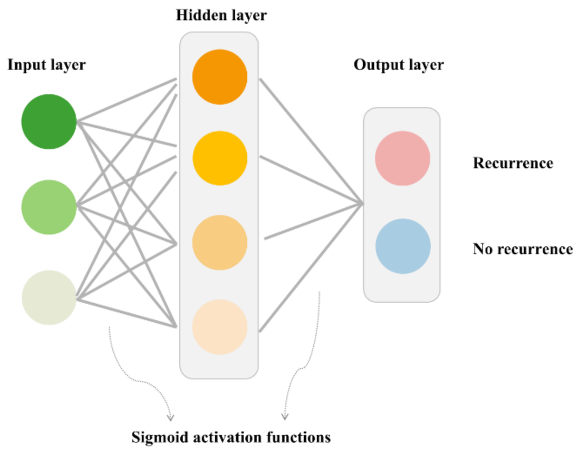


**Supplementary Figure 2.** The structure of the artificial neural network. The artificial neural network model consists of an input layer, hidden layers, and an output layer. In this study, clinicoradiological features and radiomics features serve as the inputs to the neural network, while tumor recurrence is the output of the neural network. Sigmoid activation functions were employed in the input-hidden layer and hidden-output layer.

1. Guan Z, Li Z-W, Yang D, et al (2023) Small arteriole sign: an imaging feature for staging T4a colon cancer. Eur Radiol 34:444–454. https://doi.org/10.1007/s00330-023-09968-4
